# Biomass-Derived Nitrogen-Doped Carbon Aerogel Counter Electrodes for Dye Sensitized Solar Cells

**DOI:** 10.3390/ma11071171

**Published:** 2018-07-09

**Authors:** Mira Tul Zubaida Butt, Kathrin Preuss, Maria-Magdalena Titirici, Habib ur Rehman, Joe Briscoe

**Affiliations:** 1 Department of Chemistry and Chemical Engineering, Syed Babar Ali School of Science and Engineering, Lahore University of Management Sciences, Opposite Sector U, DHA, Lahore 54792, Pakistan; 13130004@lums.edu.pk; 2 School of Engineering and Materials Science and Materials Research Institute, Queen Mary University of London, Mile End Road, London E1 4NS, UK; k.preuss@qmul.ac.uk (K.P.); m.m.titirici@qmul.ac.uk (M.-M.T.)

**Keywords:** doped carbon aerogel, hydrothermal carbonization, counter electrode, photovoltaic devices, nanoparticles

## Abstract

Dye sensitized solar cells have emerged as an attractive alternative to conventional solar cells due to their easy processing and the abundance and low cost of their materials. However, the counter electrode in these cells employs platinum which significantly impacts their cost. Here, we report biomass-derived, nitrogen-doped carbon aerogel as an effective alternative to conventional platinum-based counter electrodes for dye sensitized solar cells. A stable suspension of biomass-derived, nitrogen-doped carbon aerogel was prepared in DMF by using oleylamine as a binder. The nitrogen-doped carbon aerogel electrode was annealed at different temperatures, and its impact on photovoltaic performance is investigated. I-V measurements confirm that the annealing temperature substantially enhances the photovoltaic parameters of these devices; these enhancements are linked to the removal of the organic binders. Electrochemical impedance spectra of the counter electrodes confirm that removal of oleylamine in nitrogen-doped carbon aerogels reduces the series resistance of the resulting electrodes. The power conversion efficiency of the solar cells from optimized nitrogen-doped carbon aerogel exhibited comparable efficiency to that of a cell fabricated using a platinum-based counter electrode. This study demonstrates the potential of biomass-derived carbon aerogels as a cheap and sustainable replacement of platinum in DSSCs.

## 1. Introduction

Dye-sensitized solar cells (DSSCs) have been investigated as alternatives to current photovoltaic technologies due their low cost, simple manufacturing process, and potential for high efficiency [1,2,3,4]. Several strategies have been proposed to further lower the cost of these devices to boost their commercial potential. The counter electrode plays an important role in catalyzing the reduction of redox mediators in DSSCs [5]. For high efficiency DSSCs, the counter electrode should be conductive [6] and possess good catalytic properties [7]. However, there is often a trade-off between these two parameters. The catalytic efficiency of an electrode is generally linked to the available surface area; therefore, increasing the catalyst’s available area is an appealing strategy to enhance its activity. One way to increase this area is through reducing the particle size of the catalyst. The conductivity of a catalyst, on the other hand, is adversely impacted by the enhanced grain boundaries [8] from smaller particles. Conventional high-efficiency DSSCs use platinum as a counter electrode, which serves as a means of transporting electrons from the external circuit to the triiode/iodide redox couple, which in return regenerates the oxidized dye [9,10]. Since platinum is an expensive metal, it greatly hampers the large-scale production of these devices. In order to address this issue, many cheap and viable alternatives have been investigated in the recent past [11]. In this regard, carbonaceous materials have gained much attention due to their high electronic conductivity, low cost, and ability to efficiently reduce triiodide in the redox electrolyte [2]. Carbon-based materials such as graphene [12], CNTs [13], carbon black [2], fullerene [14], and mesoporous carbon [15] have been successfully employed to enhance the catalytic activity of the counter electrode.

Composites of carbon with other organic/inorganic materials have proven to be equally efficient when used as counter electrodes in DSSCs. Ali et al. modified the surface of carbon nanotubes with cobalt selenide nanoparticles, and achieved enhanced photoelectric conversion efficiency (6.42%) as compared to conventional platinum-based counter electrodes (5.42%) [16]. Battumur et al. incorporated a carbon nanotube-graphene composite as a platinum alternative, and gained improved photovoltaic performance [17]. Similarly, when a TiN composite with conductive carbon black was used as a counter electrode in DSSCs, high catalytic reduction of the electrolyte was achieved in comparison to platinum [18]. Due to the high surface area (up to 1100 m^2^/g) and controllable pore structure, ultrathin carbon aerogels have a good potential to effectively catalyze the reduction of the electrolyte in a solar cell. Carbon aerogels are electrically conductive (25–100 S cm^−1^), with high effective surface areas [19]. A composite of a mesoporous carbon aerogel with PTFE polymer was used on a flexible stainless steel conductive substrate as a counter electrode to achieve power conversion efficiency of 9.06%, compared to 9.14% for platinum-based counter electrodes [20]. In addition, nitrogen doping of carbon materials can enhance functionality, including for photovoltaic applications [21,22]. Nitrogen-doped porous carbons have also been used as DSSC counter electrodes; material synthesized by pyrolysis and alkali activation of melamine formaldehyde resin with surface area 1302 m^2^ g^−1^ achieved a power conversion efficiency of 6.9% when used as a counter electrode [23]. Zhu et al. synthesized nitrogen-doped carbon microspheres with a multistep microwave assisted method (surface area 1630 m^2^ g^−1^) as a counter electrode in DSSCs, achieving a PCE of 6.28% [24].

The above literature demonstrates an excellent potential of both carbon aerogels and nitrogen-doped carbons for counter electrodes in DSSCs. However, despite being a highly-abundant element, most carbon materials are derived from fossil-fuel sources, contributing to the depletion of this finite resource. It would therefore be of considerable benefit to draw on the vast resource of renewable carbon material (biomass) which is available in nature. In this work, we used a nitrogen-doped carbon (N-dC) aerogel as an efficient platinum alternative, which was prepared via an environmentally-friendly process known as hydrothermal carbonization using biomass-derived precursors [25]. Compared to other carbon-based counter electrodes, often produced from toxic chemicals and under harsh conditions, the approach used here is highly sustainable and easy to reproduce. Furthermore, DSSC assembled using a counter electrode from N-dC exhibited a light-to-current conversion efficiency close to 90% of that of a platinum electrode in the same configuration.

## 2. Materials and Methods

### 2.1. Chemicals and Materials

FTO as a transparent conducting oxide was supplied from Solaronix (Aubonne, Switzerland), having a surface resistivity of 15 Ω/sq. Titania particles (P25) for mesoporous titania paste and titanium butoxide as a precursor for compact titania layer were purchased from Sigma Aldrich (St. Louis, MO, USA). N719 dye (Ruthenizer 535-bis TBA, Solaronix, Aubonne, Switzerland), iodolyte HI-30 as an electrolyte and chloroplatinic acid (platisol) for platinum counter electrode were obtained from Solaronix. d-(+)-glucose and lyophilised ovalbumin from chicken egg white were purchased from Sigma Aldrich and used without further purification.

### 2.2. Preparation of Photoanode

FTO substrates were cleaned with detergent and sonicated in acetone and isopropanol for 15 min. Prior to coating, the FTO glass was heated at 120 °C for 10 min to remove any moisture on the substrate. A 5% titanium butoxide solution in isopropanol was used to spin coat a compact titania layer onto a FTO substrate at 3000 rpm. The substrate was annealed at 500 °C for one hour. A mesoporous titania solution was prepared by adding 0.7 g of titania P25 particles to 1 mL of methanol; this mixture was stirred overnight. A 7 µm film of mesoporous titania layer composed of around 20 nm TiO_2_ particles was spin coated at 2000 rpm for 30 s onto the compact titania layer and annealed again at 500 °C for one hour. Subsequently, the titania-based photoanode was immersed in a 40 mM aqueous solution of TiCl_4_ at 70 °C for 30 min and then heated at 500 °C for 1 h. 

### 2.3. Synthesis of N Doped Carbon Aerogel

A nitrogen-doped carbon aerogel was synthesized as described by Preuss et al. [26]. Briefly, 1.5 g of d-(+)-glucose and 0.3 g of lyophilised ovalbumin from chicken egg white were dissolved in 13.5 g deionized water by sonicating for 30 min. The mixture was prepared in a glass vial, which was placed in a Teflon inlet and transferred to a stainless steel autoclave (Parr Instruments, Moline, IL, USA). Hydrothermal carbonization was carried out at 180 °C for 5.5 h. After the hydrothermal carbonization, the sample was washed by stirring in deionized water for 24 h, followed by vacuum filtration and freeze drying for 24 h. Subsequently, the dried powder was placed in a ceramic crucible covered with a ceramic lid, and placed in a Carbolite tube furnace, followed by 15 min of flushing with N_2_. The sample was then heated under an N_2_ atmosphere at a heating rate of 7 °C/min up to 1000 °C, with a dwell time of 2 h, before subsequent cooling to room temperature.

### 2.4. Preparation of Carbon Counter Electrode

A 3% solution of N-doped carbon suspension was prepared in 1 mL of DMF. In order to stabilize the suspension, 0.15 g of oleylamine (OA) was added. The suspension was stirred for 3 h prior to coating onto a FTO substrate. N-dC counter electrodes were spin coated at 500–3000 rpm onto FTO glass slides for 30 s. These electrodes were annealed at either 200 °C or 450 °C for one hour under an inert atmosphere with a ramp rate of 10 °C/min.

### 2.5. Preparation of Platinum Electrode

For platinum, a solution of chloroplatinic acid (H_2_PtCl_6_) was spin coated at 1000 rpm for 30 s onto a pre-cleaned FTO substrate and annealed in air at 450 °C for 30 min.

### 2.6. Assembly of the Device

The photoanode was dipped in a 0.5 mM ethanolic solution of ruthenium based (N719) dye for 24 h in the dark. Afterwards, the dye-coated substrates were washed with absolute ethanol to remove excess dye. The electrolyte was added and sealed between the photo and counter electrodes. Cellophane tape was used to seal the electrolyte within the cell. 

### 2.7. Characterization and Device Testing Techniques

The morphology of the nitrogen-doped carbon aerogel was measured using an FEI-Inspect F field emission scanning electron microscope and JEOL JEM-2010 high resolution transmission electron microscope. The surface area and pore sizes for the nitrogen-doped carbon aerogel were determined with Brunauer-Emmett-Teller (BET) theory and Density Functional Theory (NLDFT), respectively, using Nitrogen absorption and desorption isotherms obtained at 77 K with a Quantachrome Nova 4200e. The J-V curves of the devices were measured with a Keithley 2400 Source Meter under simulated AM 1.5 G solar light irradiation from a Solar Simulator from Newport class ABB and in the dark. The intensity of the solar light illumination was measured using a standard silicon cell. The electrochemical impedance spectra (EIS) of symmetrical cells made by sandwiching together two counter electrodes and filling with electrolyte were measured using a Gamry Instrument Interface 1000 in a frequency range from 0.01 Hz to 100 kHz, with 10 mV amplitude, in the dark.

## 3. Results and Discussion

The N-dC aerogel material was synthesized by simple hydrothermal carbonization using the biomass-derived materials glucose and chicken egg ovalbumin. Details are given in the Experimental Methods section, with full synthesis details and extensive characterization of the material reported elsewhere [26]. The elemental composition of the doped carbon showed 3.3 wt % N, 92.3 at % C and 4.4 at % O by detailed XPS spectra and peak position analysis, confirming the formation of N-doped carbon material [26]. Dye-sensitized solar cells were constructed with both N-dC aerogel and a Pt counter electrode (CE) as reference.

**Figure 1:**
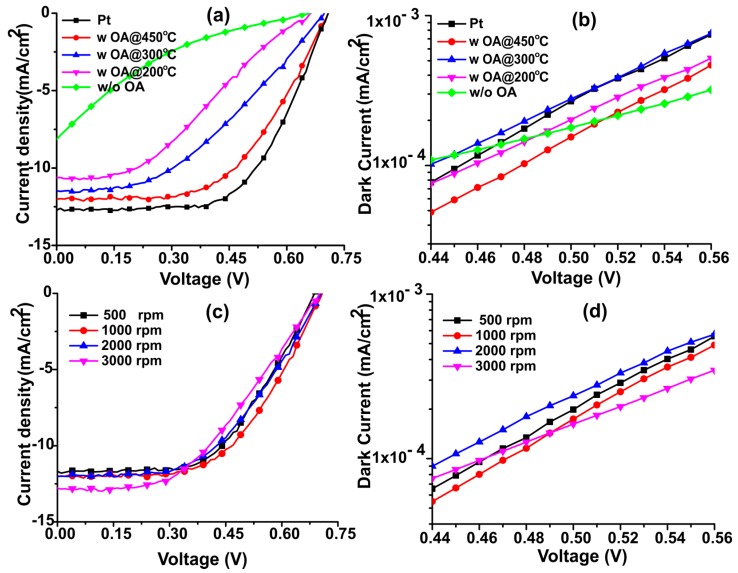
Photocurrent density-voltage (J-V) curves of solar cell devices fabricated using platinum and N-dC counter electrodes (**a**) annealed at different temperatures, and (**c**) coated at various spin speeds, (**b**,**d**) dark current plots of respective photovoltaic devices. All the J-V curves were measured on cell areas of 0.25 cm^2^ under 1 sun illumination.

The formation of a stable carbon suspension in any organic solvent has always been a challenging task. We introduced oleylamine ((Z)-Octadec-9-enylamine (OA)) as a binder to make a stable N-dC aerogel suspension in DMF. The hydrophobic part of oleylamine helps achieve stabilization of N-dC, while the amine group improves solubility in DMF. This modification significantly improved film quality and the overall photovoltaic performance of resulting cells. Cells prepared without OA performed as poorly as cells fabricated with bare FTO as the counter electrode (Figure S1). The reason for their low performance can be linked to the poor film formation characteristics of N-dC suspensions. Carbon particles during film deposition from N-dC solution without OA were sparsely spread in large domains or agglomerates due to their limited adhesion with the substrate. With the introduction of OA, however, stability of the suspension and adhesion of the carbon particles with FTO was greatly improved, resulting in uniform, stable, and good quality films. In order to investigate the impact of the oleylamine (OA) on the photovoltaic parameters of these devices, cells were fabricated using CEs with and without oleylamine. The photovoltaic performance of these devices is shown in Figure 1a and statistical photovoltaic parameters are summarized in Table 1 and Table 2. It is clear from this data that the current density, fill factor, and the efficiency of devices fabricated with N-dC electrodes having no OA decreased considerably, because the carbon nanoparticles were washed off as soon as the electrolyte came into contact with the CE. This explains why the device performance for the counter electrode without any OA was similar to the performance of a device fabricated by using bare FTO as a counter electrode (See Figure S1 and Table S1).

It can be seen from Figure 1b and Table 1 that the performance of the DSSCs fabricated using N-dC aerogel as a counter electrode is greatly affected by the annealing temperature. The photovoltaic parameters of these devices were significantly enhanced when the carbon film containing OA was annealed at 450 °C, instead of 300 °C or 200 °C. Annealing at 450 °C removed any excess OA from the carbon film, as shown from previous thermogravimetric analysis [27]. As is clear from Table 2, solar devices fabricated with a N-dC electrode heated at 200 °C show a higher series resistance (Rs), i.e., 96.23 Ω, compared to the 89.19 Ω when annealing temperature was increased to 300 °C. This value was reduced to 84.81 Ω when the annealing temperature was further increased to 450 °C. In fact, the resistance value of the N-dC sample annealed at 450 °C approaches that of platinum (78.23 Ω). The high series resistance of N-dC electrodes annealed at 200 °C can be linked to the presence of long carbon chains of organic OA molecule in the binder structure that act as insulating barriers within the carbon film, and hence, restrict the conduction of electrons across the electrode, resulting in increased series resistance. A higher annealing temperature ensures the removal of insulating groups of OA, and thus helps in reducing series resistance. Our best device from N-dC aerogel produced a current density (J_sc_) of 12.01 mA/cm^2^, open circuit voltage (V_oc_) of 0.70 V, and a fill factor of 0.56, resulting in device efficiency of 4.68%, while the corresponding photovoltaic parameters of the best platinum electrode device, respectively, are J_sc_ 12.71 mA/cm^2^, V_oc_ 0.71, FF 0.59 and PCE 5.35%. The average power conversion efficiencies of N-dC based cells at 200, 300 and 450 °C are 2.39 ± 0.20%, 3.47 ± 0.16% and 4.47 ± 0.21%, respectively. A higher annealing temperature has led to substantial improvements in J_sc_, FF, and PCE. The improved photovoltaic performance of samples annealed at higher temperatures can be linked to the porous structure of N-dC aerogel materials (surface area of 158 m^2^ g^−1^, Supporting Information Figure S2b), coupled with reduced series resistance by the removal of insulating groups of OA at higher temperatures, and increased defect sites brought about the nitrogen-doping [28,29,30]. The average efficiency of carbon-based films (4.47 ± 0.21%) annealed at 450 °C was still a little lower than that of conventional platinum based counter electrode (5.08 ± 0.24%) (see Table 1). The difference in device performance of the N-dC electrode compared to that of a platinum cell could be due to the slightly enhanced series resistance and still not optimal adhesion of the aerogel layer to the FTO, which can significantly impact the quality of the diode [31]. To further probe the impact of thermal annealing on the diode quality (recombination kinetics) of resulting photovoltaic devices, dark current vs. potential curves for these devices are plotted in Figure 1b. It is clear from this figure that the slopes of Pt and N-dC devices annealed at 450 °C show more or less similar values. Devices annealed at lower temperatures, however, show a gradual decrease in their slopes with decreasing temperatures. These lower slopes can be linked to the enhanced dark currents which arise due to poor quality of diodes and poor contacts between the electrode layer and the underlying conducting substrates. Additionally, higher dark currents lead to lower output power, as weaker diodes give way to higher recombination. The lowest slope and PCE is observed for devices with no coating at all. This can be linked to the poor catalytic ability and highest recombination reactions in these devices.

The effect of N-dC film thickness on device efficiency was also investigated by varying the spin speeds during carbon aerogel deposition. Figure 1c gives I-V scans of the best devices realized at four spin speeds (500, 1000, 2000 and 3000 rpm), and Table 2 lists the average performance parameters of eight devices at each temperature. It can be seen from Table 2 that the current density and open circuit voltage was independent of the spin speed within error; however, FF of the devices was reduced from 0.56 ± 0.01 to 0.50 ± 0.02 as the spin speed increased from 500 rpm to 3000 rpm (reducing the film thickness). This confirms that generally thinner films yield higher efficiencies. At lower spin speed (500 rpm), PCE was slightly reduced compared to that of a 1000 rpm device. This could be due to the fact that thicker films offer higher series resistance and poor adhesion, or because the surface coverage was not complete at this very low spin speed. The decrease in efficiency for thinner films, despite their lower series resistance, may be linked to the poor adhesion and limited surface coverage of these carbon films. Poor surface coverage can lead to enhanced recombination and dark current. Figure 1d shows dark current scans of these devices. Devices from both 500 and 1000 rpm deposited films show the highest but similar slope values. However, these values constantly decrease with further increase in deposition speeds; this can be attributed to the enhanced recombination rates due to incomplete surface coverage. Furthermore, the uniformity of the thinner film was reduced, which resulted in the decrease in device FF.

**Figure 2:**
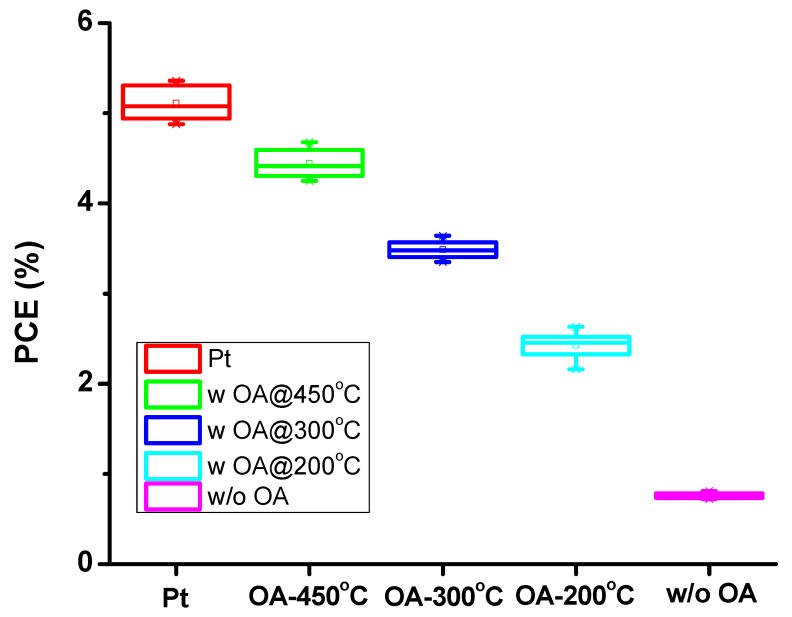
Box plots showing the upper, lower and mean values of the efficiencies obtained by using platinum and carbon based counter electrodes in DSSCs. For each set of devices, eight cells were prepared and all devices were tested for exposure area of 0.25 cm^2^ under 1 sun illumination.

In order to look into the reproducibility of these devices, eight sets of devices were fabricated; the upper, lower, and mean values of the efficiencies of all the DSSCs devices fabricated using platinum- and carbon-based counter electrodes are shown in a box plot in Figure 2. The results presented in Figure 2 and Table 2 show that there is a marked improvement in the performance of the CE when heated at 450 °C compared to 200 °C. It is clear from the box plot that both the average and the highest efficiencies of devices fabricated with N-dC electrodes increased when annealing temperatures of these devices was increased from 200 °C through 300 °C to 450 °C. When the temperature of N-dC electrode was increased to 450 °C, a marked improvement in both highest (4.68%) and average efficiency (4.47 ± 0.21%) was observed. This efficiency enhancement—at higher annealing temperature—of N-dC electrode devices can be linked to the decrease in series resistance, increase in catalytic reduction reaction of redox couple due to higher surface area, and better chemical reactivity, along with improved charge collection efficiency (discussed below).

**Figure 3:**
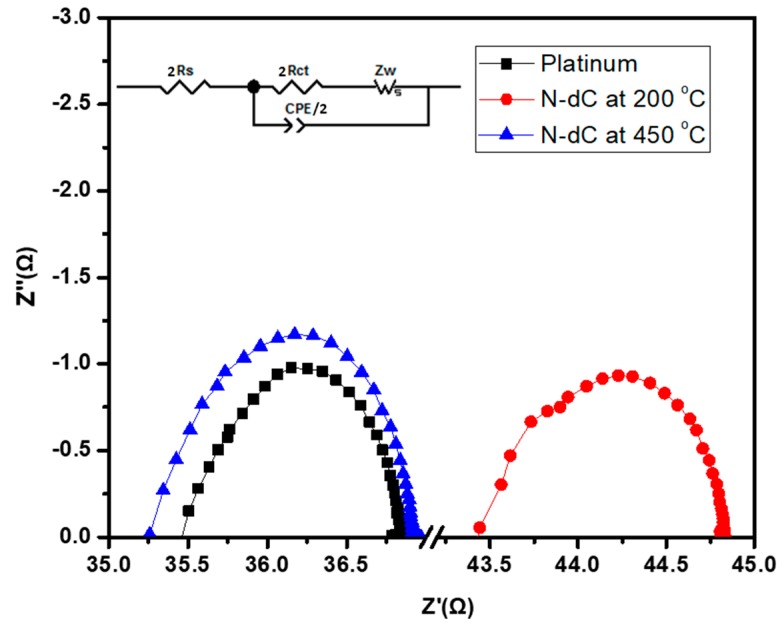
Nyquist plot of symmetrical cells with two identical electrodes of conventional platinum, and N-dC aerogel, annealed at 200 and 450 °C, working as counter electrode. The plots were taken in the frequency range of 0.01 Hz to 1 MHz with 10 mV amplitude in the dark. All devices were biased at Voc. The inset of the figure shows the equivalent circuit diagram of the symmetrical cells used to fit the impedance data; R_s_: series or internal resistance, R_ct_: charge transfer resistance, Z_w_: Diffusion Impedance, CPE: constant phase element.

In order to investigate the origin of performance differences between the CEs treated under different conditions, the charge transfer mechanism between the counter electrode and electrolyte was investigated using electrochemical impedance spectroscopy (EIS). In order to study the charge transfer between counter electrode and electrolyte interface and catalytic activity of N-dC electrode, a symmetric cell was fabricated by sandwiching the electrolyte between two identical electrodes (FTO/N-dC/Electrolyte/N-dC/FTO or FTO/Pt/electrolyte/Pt/FTO). Figure 3 shows the typical Nyquist plots of such symmetrical cells using platinum- and carbon-based electrodes; the inset shows the equivalent circuit diagram used to fit EIS data. Each semicircle shows the interfacial charge transfer resistance (R_ct_) between the counter electrode and electrolyte and interfacial capacitance (CPE) at the electrode/electrolyte interface, which corresponds to the catalytic ability of the electrode to reduce the electrolyte (I_3_^−^ to I^−^) [32]. The charge transfer resistances of the carbon-based counter electrodes (0.83 Ω and 0.65 Ω) annealed at 450 °C and 200 °C are comparable to that of the platinum control (0.7 Ω) (see Table 3), reflecting its relatively high catalytic performance. Similar results have been reported by Lee group for a multiwalled, carbon nanotube-based counter electrode [33]. The series resistance (R_s_) describes the resistance through the electrode material, and can be a measure of electrode layer bonding with the FTO [24]. The constant phase element (CPE-T) corresponds to the capacitance of the electrolyte/electrode interface, while CPE-P reflects how close the CPE-T is to the true capacitance (Cμ). A CPE-P generally has values ≤1, whereCPE-T value of 1 represents the true capacitance. A CPE-P value lower than 1 suggests a deviation of CPE from ideal capacitance, and is called *ideality factor*. The lower CPE-P value can be linked to the rougher or more porous electrode surfaces [34]; the higher the roughness, the lower the CPE-P. It is clear from Figure 3 and Table 3 that the series resistance of the N-dC-based electrode reduces from 21.73 Ω to 17.63 Ω, while the charge transfer resistance slightly increases from 0.70 Ω to 0.83 Ω when the annealing temperature was increased from 200 °C to 450 °C. A similar trend is observed for both CPE-T and ideality factor for electrodes at two temperatures. CPE-T and CPE-P values of a symmetric cell obtained from N-dC electrodes annealed at 200 °C agrees really well with the Pt cell, while both these values are slightly lower for the 450 °C annealed sample. A lower ideality factor of the 450 °C annealed electrode over that of the 200 °C electrode could be due the increased porosity/roughness of the 450 °C annealed sample, due to the complete removal of OA. Capacitance values calculated from this data show a similar trend, with minimum capacitance for the 450 °C annealed sample. The lowest capacitance, coupled with the enhanced surface area of the 450 °C annealed electrode can be linked to the increased catalytic reduction reactivity of this electrode. The charge collection efficiencies of these electrodes were also calculated, and the collection efficiency of the 450 °C anneal sample was found to be ~17% higher than that of Pt electrode. The sample annealed at 200 °C showed about a 24% lower collection efficiency when compared to that of the Pt electrode. This confirms that the lower performance of the CE annealed at 200 °C was due to additional series resistance and charge collection efficiency resulting from oleylamine that remained in the film.

**Figure 4:**
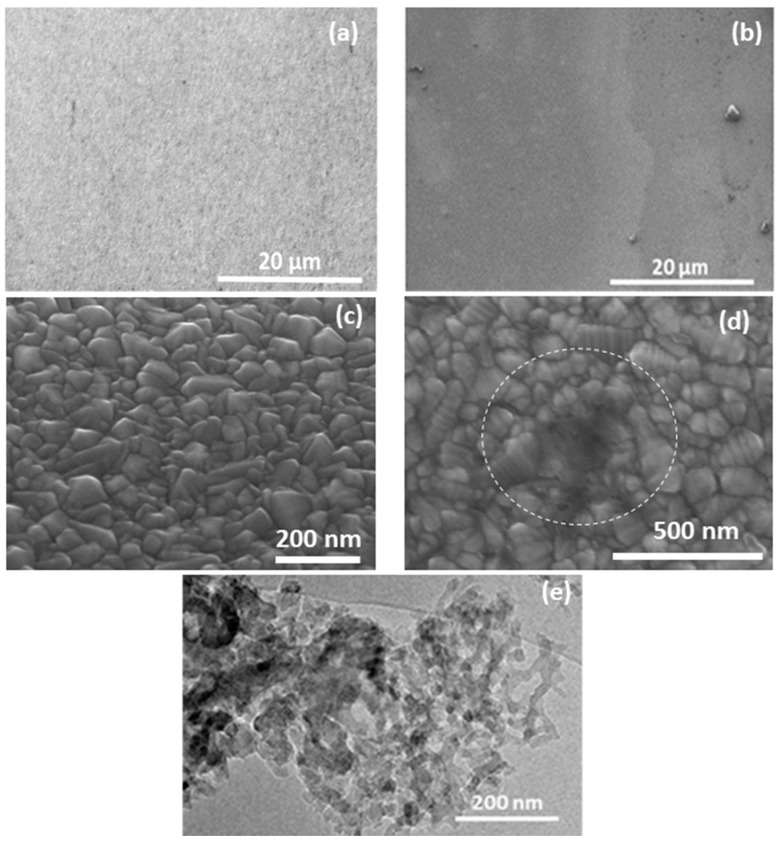
SEM images (**a**) and (**c**) of bare FTO and (**b**) and (**d**) of N-dC aerogel at different magnifications heated at 450 °C. (**e**) High magnification TEM image of the carbon aerogel pyrolysed at 1000 °C.

The SEM images of bare FTO and carbon aerogel film deposited on FTO substrate are presented in Figure 4. A fairly uniform N-dC layer covering the FTO surface can be seen as a variation in surface contrast in Figure 4b, compared to the uniform, uncoated surface in Figure 4a. Figure 4d presents an enlarged view of FTO coated with thin carbon structure. Although it is very difficult to image due the low thickness—no more than a few nm thick—of the overlaying crystalline FTO surface and low interaction with the electron beam, some areas of thicker carbon can be seen, as circled in Figure 4d, which are not present on the uncoated FTO (Figure 4c). To further investigate the morphology of N-dC materials, a TEM analysis was performed on the aerogel by directly depositing it onto conductive grids. The TEM micrograph confirms the nanoscale nature of the carbon nanogels (Figure 4e). The combined evidence of the TEM and SEM analysis implies that nanogels have been uniformly coated onto the FTO substrates in films only a few nm thick, and that these coated substrates act as an efficient catalyst when used as counter electrode in DSSCs. Additionally, the high surface area (158 m^2^/g—see Supporting Information Figure S2) of the carbon aerogel also contributes to the high catalytic activity of these electrodes.

## 4. Conclusions

This work highlights the use of a biomass-derived carbonaceous material as an effective alternative to expensive platinum counter electrodes in dye-sensitized solar cells. The nitrogen-doped carbon aerogel was synthesized from biomass-derived precursors through an environmentally-friendly and sustainable technique. A stable and homogeneous suspension was obtained using oleylamine as a binder. Annealing at high temperature increased device performance; this is linked to the removal of binder from the deposited films, which otherwise hampers device performance due to long alkyl chains causing high series resistance. The high surface area of such aerogels improved the DSSC performance by catalyzing the reduction reaction of redox mediators. The method used to synthesize the carbon aerogel allows easy tuning of surface area and pore volume/sizes by simple adjustment of precursors and reaction conditions, presenting a range of promising and inexpensive materials. Future work will aim to increase the surface area of the aerogel, which would be expected to further increase device efficiency. N-dC counter electrodes, due to their comparable performance to Pt-based counter electrodes in DSSCs, have excellent potential as a low-cost and sustainable replacement of expensive platinum electrodes in DSSCs.

## Figures and Tables

**Table 1:** Effect of annealing temperature and binder on the photovoltaic parameters of the DSSCs prepared using platinum and N-dC aerogel as counter electrodes. The platinum precursor and carbon suspensions were spin coated at 1000 rpm. All measurements were performed on 0.25 cm^2^ area cells using light intensity of 100 mW/cm^2^. The reported values represent the averages of eight samples.

Samples	Annealing Temperature (°C)	Current Density (mA/cm^2^)	Open Circuit Voltage (V)	Fill Factor	Efficiency (%)	Rs (Ω)
Platinum	450	12.04 ± 0.58	0.71 ± 0.02	0.58 ± 0.02	5.08 ± 0.24	78.38
N-dC with OA	450	11.62 ± 0.40	0.70 ± 0.01	0.56 ± 0.01	4.47 ± 0.21	84.81
N-dC with OA	300	11.45 ± 0.44	0.69 ± 0.01	0.44 ± 0.02	3.47 ± 0.16	89.19
N-dC with OA	200	10.85 ± 0.56	0.67 ± 0.02	0.33 ± 0.03	2.39 ± 0.20	96.23
N-dC w/o OA	200	8.06 ± 0.20	0.66 ± 0.03	0.15 ± 0.01	0.78 ± 0.03	145.01

**Table 2:** Effect of different spin speeds on the photovoltaic parameters of DSSC devices prepared from N-dC counter electrodes heated at 450 °C. All electrodes were prepared with the same amount of binder (oleylamine). All measurements were performed on 0.25 cm^2^ area cells using light intensity of 100 mW/cm^2^. The reported values represent the averages of eight values.

Spin Speeds (rpm)	Current Density (mA/cm^2^)	Open Circuit Voltage (V)	Fill Factor	Efficiency (%)	Rs (Ω)
500	11.42 ± 0.36	0.68 ± 0.01	0.56 ± 0.01	4.40 ± 0.13	86.09
1000	11.62 ± 0.4	0.70 ± 0.01	0.56 ± 0.01	4.47 ± .21	84.81
2000	11.59 ± 0.28	0.70 ± 0.01	0.54 ± 0.02	4.31 ± 0.19	84.06
3000	11.71 ± 0.41	0.70 ± 0.01	0.50 ± 0.02	4.01 ± 0.26	80.01

**Table 3:** Impedance parameters of symmetric cells with conventional platinum and N-dC-based counter electrodes annealed at two different temperatures.

Sample	2R_ct_ (Ω)	2R_s_ (Ω)	CPE-T (µF)	CPE-P	C/2 (µF)	Rs* (Ω)
Pt	0.70	17.73	0.92	0.86	1.60	78.38
N-dC (450 °C)	0.83	17.63	0.84	0.80	1.38	84.81
N-dC (200 °C)	0.65	21.73	0.91	0.85	1.56	96.23

Rs* = series resistance values of DSSCs calculated from I-V curves.

## Supplementary Materials

The following are available online at http://www.mdpi.com/1996-1944/11/7/1171/s1, Figure S1: J-V curves of the devices fabricated by using FTO and carbon suspension without OA as counter electrodes. Figure S2: (a) The pore size distribution (b) surface area of the nitrogen-doped carbon aerogel determined with Density Functional Theory (NLDFT) and Brunauer-Emmett-Teller (BET) theory, respectively, using Nitrogen absorption and desorption isotherms obtained at 77 K with a Quantachrome Nova 4200e. Table S1: Photovoltaic parameters of FTO and N-dC aerogel without OA as a counter electrode in dye sensitized solar cells under 1 sun illumination with 0.25 cm^2^ device area.
