# Supplementary material for: Biomass-Derived Nitrogen-Doped Carbon Aerogel Counter Electrodes for Dye Sensitized Solar Cells
Source: Materials (Basel). 2018 Jul 9;11(7):1171. doi: 10.3390/ma11071171 (PMC6073752; doi:10.3390/ma11071171)
Supplement: Supplementary file 1 [file materials-11-01171-s001.zip › materials-320300-SI.pdf]

Supporting Information

Biomass-derived Nitrogen-doped Carbon Aerogel Counter Electrodes for Dye Sensitized Solar Cells

Mira Tul Zubaida Butt,[1,2] Kathrin Preuss,[2] Maria-Magdalena Titirici,[2] Habib ur Rehman[*,1] and Joe Briscoe[*,2]

[1]Department of Chemistry and Chemical Engineering, Syed Babar Ali School of Science and Engineering, Lahore University of Management Sciences, Opposite Sector U, DHA, Lahore - 54792 Pakistan.

[2]School of Engineering and Materials Science and Materials Research Institute, Queen Mary University of London, Mile End Road E1 4NS, London United Kingdom.

, Queen Mary, University of London, Mile End Road E1 4NS, London United Kingdom.

*j.briscoe@qmul.ac.uk, habib.rehman@lums.edu.pk

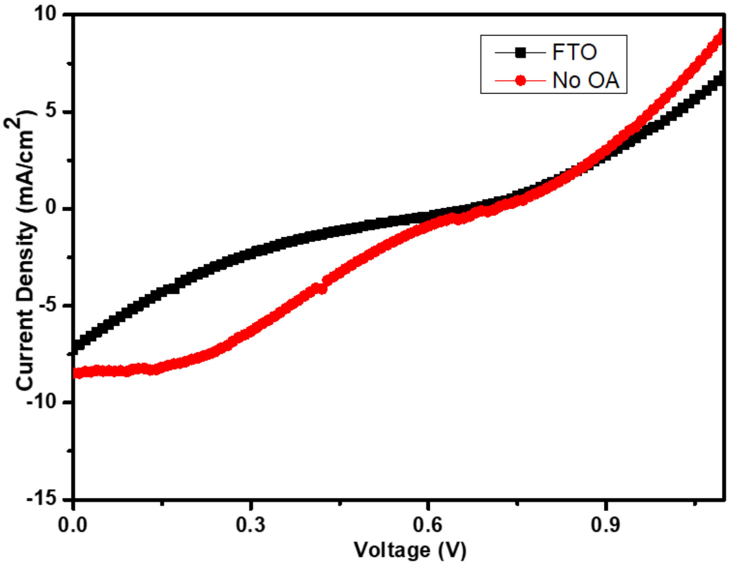

Figure S1 J-V curves of the devices fabricated by using FTO and carbon suspension without OA as counter electrodes.

Table S1 Photovoltaic parameters of FTO and N-dC aerogel without OA as a counter electrode in dye sensitized solar cells under 1 sun illumination with 0.25 cm^2 device area.

Cell Type	Current Density	Open Circuit Voltage	Fill Factor	Efficiency
	(mA/cm^2)	(V)		(%)
FTO	7.84	0.68	0.15	0.78
N-dC w/o OA	8.47	0.69	0.28	1.70

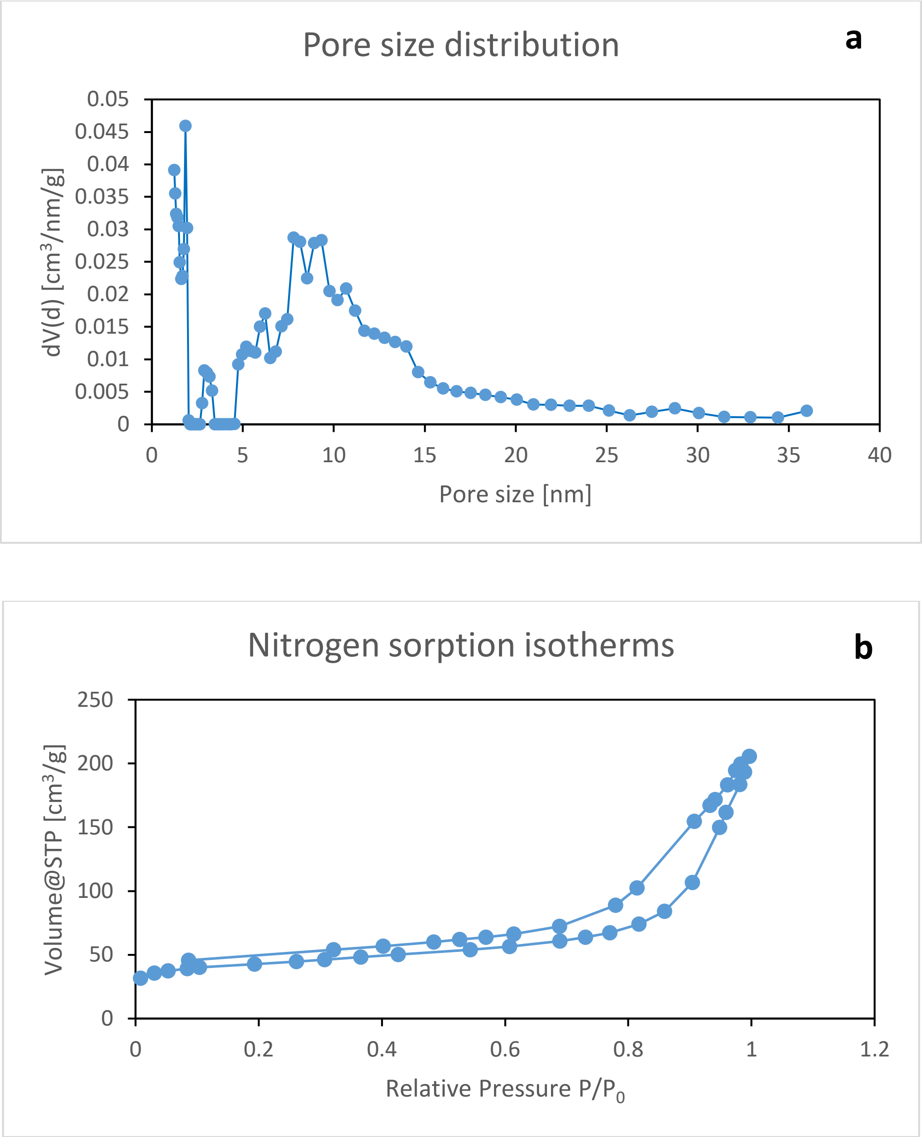

Figure S2 a) The pore size distribution b) surface area of the nitrogen-doped carbon aerogel determined with Density Functional Theory (NLDFT) and Brunauer-Emmett-Teller (BET) theory, respectively, using Nitrogen absorption and desorption isotherms obtained at 77 K with a Quantachrome Nova 4200e.

Surface area 158 m^2/g
Total pore volume 0.275 cm^3/g
Mesoporous volume 0.231 cm^3/g
Microporous volume 0.044 cm^3/g
